# Pre‐Crop Choice Shapes Nematode‐Attached Bacterial Communities Associated With Reduced *Pratylenchus penetrans* Invasion of Barley Roots

**DOI:** 10.1111/1462-2920.70179

**Published:** 2025-09-18

**Authors:** Ahmed Elhady, Xorla Kanfra, Shimaa Adss, Holger Heuer

**Affiliations:** ^1^ Julius Kühn‐Institute (JKI)—Federal Research Centre for Cultivated Plants Institute for Epidemiology and Pathogen Diagnostics Braunschweig Germany; ^2^ Darwin21 Desert Research Initiative, Biological and Environmental Science and Engineering Department King Abdullah University of Science and Technology Thuwal Saudi Arabia; ^3^ Department of Plant Protection, Faculty of Agriculture Benha University Benha Egypt

**Keywords:** bacterial attachment, cuticle, microbiome, nematode, pre‐crop, rhizosphere, suppression

## Abstract

Soil microbiomes play a crucial role in plant–parasitic nematode suppression; however, the influence of plant–soil interactions remains unclear. This study examines plant–soil feedback effects on microbiomes attached to the cuticle of *Pratylenchus penetrans* in winter barley. We tested whether bacterial drivers of nematode suppression remain conserved across plant hosts or exhibit host specificity. Surface‐sterilised *P. penetrans* were baited in different soils and rhizospheres, and their attached bacterial communities were analysed. Fallow and rhizosphere microbiomes from reduced *P. penetrans* invasion in barley, and suppression strength varied by plant species. Only the maize and Ethiopian mustard microbiomes inhibited invasion relative to other microbiomes and to surface‐sterilised nematodes. By contrast, association with the oat microbiome did not reduce *P. penetrans* invasion of barley roots. The suppression of *P. penetrans* invasion relied on the cuticle‐associated bacteria, with maize showing a distinct assembly rich in Proteobacteria and Firmicutes. Suppressive cuticle‐associated bacteria differed between nematodes exposed to maize‐derived and Ethiopian mustard‐derived rhizosphere microbiomes from the same soil. Specific bacterial genera associated with reduced invasion included *Chryseobacterium*, *Duganella*, *Streptomyces*, *Asticcacaulis*, *Pseudomonas*, and members of Enterobacteriaceae. These results indicate that crop rotation and cover crop choices could steer nematode‐associated microbiomes toward communities that prevent root invasion.

## Graphical abstract

Plant species vary in how they shape nematode‐suppressive soil microbiomes, and these legacies influence the subsequent crop. Suppressive microbiomes contain specific bacterial taxa that attach to the nematode cuticle, leading to reduced nematode invasion into the root system of the host. In contrast, some plant species enrich microbiomes with non‐suppressive or facilitative microbes, which elevate host susceptibility to nematode infection.

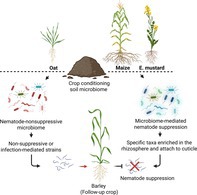

## 1 Introduction

Plant–soil feedback refers to dynamic interactions that take place between plant and soil components and influence microbial performance, soil properties, and ecosystem functions (Putten et al. 2016; Brinkman et al. 2017; Kuťáková et al. 2018). This results in inherited legacies that have either positive or negative impacts on the following crop (Detheridge et al. 2016; Lapsansky et al. 2016). Through secreted exudates, plant species create a nutrient‐rich rhizosphere, influencing soil microbial communities around their roots. These exudates include different molecules that vary between the plant species and genotypes (Badri and Vivanco 2009; Latz et al. 2016). Root exudates, host plant genetics, and root morphology collectively influence the composition of the rhizosphere microbiome by mediating the secretion and perception of key molecules between plants and associated microbes (Venturi and Keel 2016). Interestingly, plants differ in their ability to modulate the rhizosphere microbiome (Sasse et al. 2018). For instance, rhizosphere microbiomes of Arabidopsis and rice are similar to that of fallow soil (Schlaeppi et al. 2014; Bulgarelli et al. 2015), while maize and lotus have a microbial community in their rhizospheres that vastly differs from that in the fallow soil (Peiffer and Ley 2013; Zgadzaj et al. 2016).

Plant–parasitic nematodes (PPNs) pose significant economic threats worldwide. Globally, they are responsible for a substantial 12.6% of crop losses, equivalent to an annual economic impact of $216 billion (Nyaku et al. 2017). They are prevalent in soil, migrate in the soil to reach host plants, and feed on the cytoplasm of the living root cells. To complete their life cycle, they must locate the host roots, recognise and respond to plant signals, evade detection, and interact with root exudates (Bell et al. 2019). Through co‐evolution with their hosts, PPNs have acquired the ability to decipher and exchange molecular signals during early entry and even within the host tissues (Curtis 2008). Plant signals in root exudates or phytohormones are detected by chemosensory organs (amphids and phasmids) located on the head and tail of PPNs. These signal molecules can trigger rapid changes in the surface structure of PPNs (López de Mendoza et al. 2000; Akhkha et al. 2002; Curtis 2008). The surface coat that is secreted from the hypodermis comprises mainly glycoproteins, lipids, and carbohydrates (Spiegel and McClure 1995; Davies and Curtis 2011). It can be involved in microbial attachment (Danks and Davies 1993; Davies and Curtis 2011). The plant perceives components of the nematode surface (Mendy et al. 2017). Thus, changes in the surface structure before root invasion might reduce recognition by the plant (Blaxter et al. 1992), as the nematode surface coat is constantly renewed and eventually changes in response to the host within minutes (Proudfoot et al. 1993).

Nematode management is challenging because nematicides have been mostly banned, and resistant varieties or non‐host crops are often unavailable or not profitable. Novel methods with high potential and sustainability have become an urgent need. In the past, the focus was on using microbial inoculants that are commercially available and usually comprise one or several microbial species. However, their efficiency in the field is highly variable. For instance, the lack of establishment in the environment due to competition with the indigenous microbiome is a problem (Stirling 2014; Gadhave et al. 2016; Raaijmakers and Mazzola 2016). Recently, the soil microbiome emerged as a critical component in governing plant responses and shaping plant immunity to suppress pathogens (Alivisatos et al. 2015; Pieterse et al. 2016; Leach et al. 2017). Thus, it has been suggested that harnessing beneficial plant microbiomes will increase the sustainability and productivity of agriculture (Busby et al. 2017).

Monoculture sometimes enriches antagonists of plant–parasitic nematodes ( *Pasteuria penetrans* , Dactylella oviparasitica, Nematophthora gynophila) but often triggers yield decline and pest outbreaks (Hamid et al. 2017). Crop rotation tactics that include alternating host and non‐host crops can boost soil and plant health and suppress pathogens (McDaniel et al. 2014; Benitez et al. 2017; Lehman et al. 2017). Disease suppression results from complex microbial networks that include several keystone taxa. This complex ecological network could be harnessed to enhance the plant defence response to PPNs and other pathogens (Hol et al. 2010; Faust and Raes 2012; Johns et al. 2016). It was suggested that the soil quality could be modulated by targeting certain keystone microbes (Agler et al. 2016; van der Heijden and Hartmann 2016; Niu et al. 2017). In that aspect, as an agricultural practice, it was suggested that soil with disease‐suppressive properties could be transplanted to other soils to enhance their suppressiveness against pathogens (Raaijmakers and Mazzola 2016), e.g., via compost application (Olabiyi and Oladeji 2014; Coelho et al. 2021). Soils suppressive to PPNs have been described, and their microbiome has been analysed (Bent et al. 2008; Adam et al. 2014; Elhady et al. 2017). However, we still lack a clear understanding of which microbial consortia drive nematode suppression and how farmers can establish them reliably through crop diversification (Busby et al. 2017).

This study aims to understand the influence of the soil microbiome, modulated by distinct plant species, on the suppression of root‐lesion nematodes (*Pratylenchus penetrans*) in winter barley. We seek to explore the extent to which the impact of different plant species on soil bacterial community composition contributes to shaping the microbiome associated with *P. penetrans* and their invasion into the root system of the host plant. We want to explore whether these bacterial species are conserved across different plant species or exhibit host plant specificity. We attempt to identify specific bacterial species responsible for *P. penetrans* suppression. These findings could pave the way for innovative strategies in controlling plant–parasitic nematodes by considering the nematode‐associated microbiome. For instance, this might involve optimising crop rotation and growing cover crops to engineer plant‐associated microbial communities, enhancing their ability to suppress PPNs and enhance crop yield stability.

## 2 Materials and Methods

### 2.1 Effect of the Microbiome Attached to *P. penetrans* on Their Invasion Into Barley Roots

To study the role of nematode‐attached microbes in suppressing nematode invasion into host plant roots, we asked whether microbes from the rhizosphere soil of different plant species such as maize ( *Zea mays* L. cv. Colisee), Ethiopian mustard ( *Brassica carinata* cv. Cappuccino), oat ( *Avena strigosa* cv. Luxuria) or fallow soil, upon attaching to nematode cuticles, could reduce the nematode invasion rate into barley roots. Briefly, nematodes were incubated in soil suspensions of the fallow or rhizosphere of different plant species growing in a pot system in the greenhouse. Soil was collected from arable fields in Braunschweig: a sandy loamy, 1% humus, pH 6.3, field located at 52°16′21.7″ N, 10°34′02.7″ E (soil type B) (further details in Table S1). We sampled the fallow microbiome from unplanted pots and the rhizosphere microbiomes from pots planted with maize, Ethiopian mustard ( *Brassica carinata* cv. Cappuccino), or oat ( *Avena strigosa* cv. Luxurial). Two independent trials were conducted to examine microbiomes from fallow, maize, oat, and Ethiopian mustard rhizospheres and produced 24 replicates per treatment. Full methodological details appear in Section 2.2.

Barley seeds of the winter cultivar “Igri” were surface sterilised by washing them three times for 1 min per wash with sterile water. Subsequently, seeds were incubated in 1.5% sodium hypochlorite, slowly shaking at 150 rpm for 20 min. The seeds were rinsed five times with sterile water and left for 15 min to dry on the clean bench. Seeds were germinated in Petri dishes (100 mm × 15 mm) on 1% water agar for 4 days under sterile conditions. Uniformly developed seedlings were transferred to 25 mL of 0.8% Gelrite solidified medium supplemented with half‐strength Murashige and Skoog (MS) salts (Duchefa Biochemie). Each seedling was placed on an individual Petri dish and exposed to 600 nematodes either carrying their native microbiome or surface‐sterilised after recovery from rhizosphere‐ or fallow‐soil suspensions. The dishes were sealed, positioned horizontally in a climate chamber, and arranged in a randomised complete‐block design for 3 days to allow nematode penetration. After 72 h, the roots were excised, stained with 1% acid fuchsin, and the nematodes inside the tissues were counted to quantify invasion (Bybd et al. 1983).

### 2.2 Bacterial Communities Associated With the Cuticle of *P. penetrans* in Rhizospheres

#### 2.2.1 Surface‐Sterilisation

**FIGURE 1:**
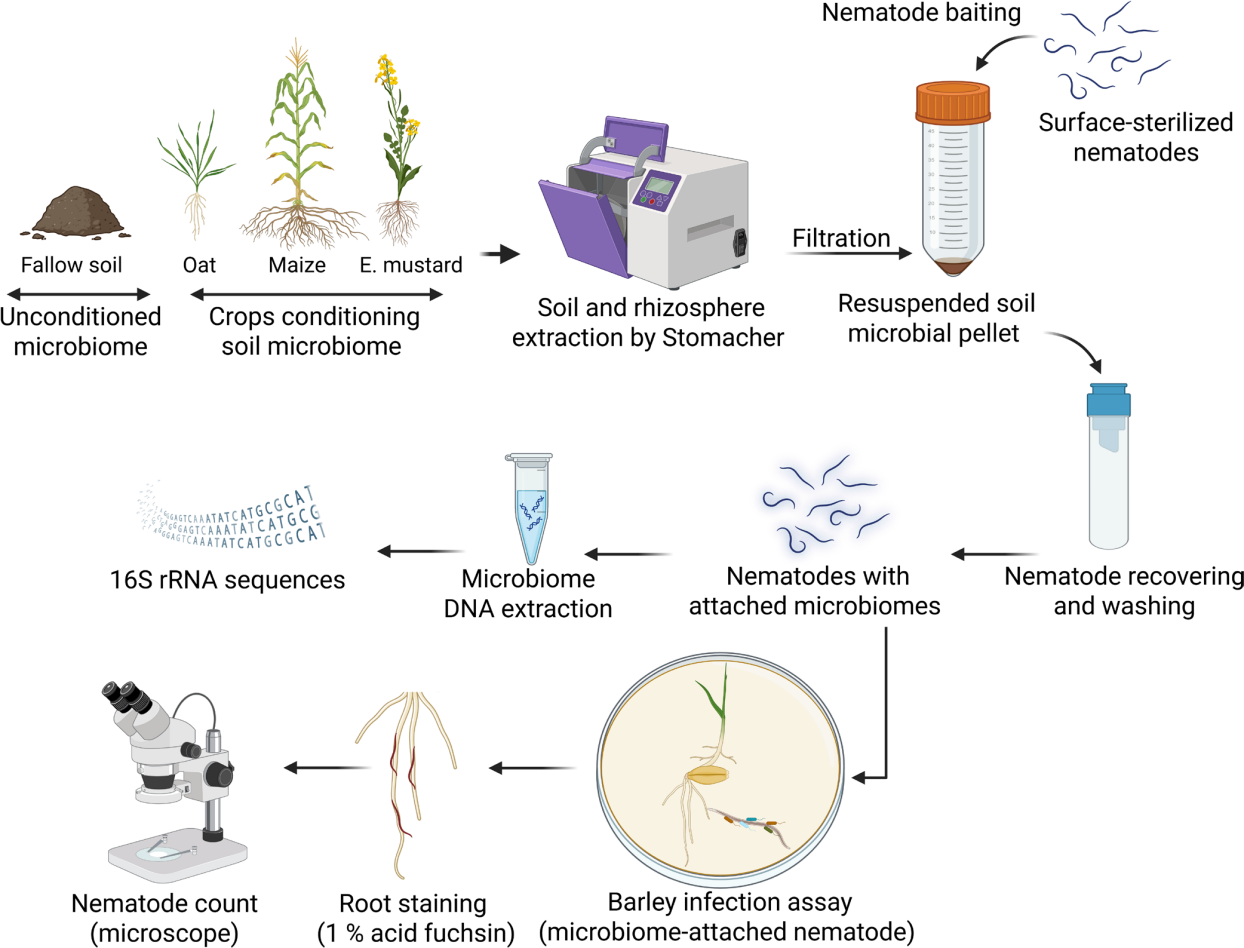
The experimental design was used to investigate the suppressive effect of soil and rhizosphere microbiomes on root‐lesion nematodes (*Pratylenchus penetrans*). The steps included microbiome production and conditioning by different plant species, extraction of soil and rhizosphere samples, and baiting surface‐sterilised nematodes in the microbial suspensions. Total DNA of nematode‐associated microbiomes was extracted, bacterial ribosomal gene fragments amplified by PCR, followed by high‐throughput sequencing of the amplicons. Barley root infection was quantified in vitro for nematodes with cuticle‐attached microbiomes from fallow and rhizosphere soils compared to surface‐sterilised nematodes. The nematodes were stained with 1% acid fuchsin and quantified under a microscope.

A population of *P. penetrans* was obtained from the Institute for Agricultural, Fisheries, and Food Research (ILVO, Belgium). Nematodes were maintained and propagated on carrot discs for 2–3 months and extracted using a Baermann funnel in a spray‐mist chamber for two weeks. The collected suspension was cleaned on the Baermann funnel to remove the remaining carrot tissues. Nematodes were surface sterilised as previously described (Elhady et al. 2018). Nematodes were placed on a 5‐μm sieve (Cell‐Trics1 filters, Sysmex, Norderstedt, Germany) on a 15 mL Falcon tube to receive the through‐flow suspension. The nematodes were then washed with 10 mL sterile tap water and treated with 0.02% HgCl_2_ for 3 min and a 200 mg L^−1^ streptomycin sulphate solution for another 3 min. The nematodes were transferred to a 50 mL Falcon tube and incubated on a rotary shaker at 150 rpm for 4 h in 5 mL of an antimicrobial solution (CellCultureGuard, AppliChem, Darmstadt, Germany). To confirm complete surface sterilisation, nematodes were plated on Reasoner's two agar (R2A) and Potato Dextrose Agar (PDA). No microbial colonies appeared after a 48‐h incubation period, and the nematodes were subsequently used for microbiome inoculation. After surface disinfection, the nematodes were washed with sterile tap water on a 5‐μm sieve and incubated overnight to recover (Figure 1).

#### 2.2.2 Nematode Baiting in Microbial Suspensions

The surface‐sterilised nematodes were incubated in fallow and rhizosphere soils, as previously shown (Elhady et al. 2021), to explore the microbiome profile of nematodes as affected by their migration through the fallow soil to the rhizosphere. Briefly, nematodes were incubated in soil suspensions of the fallow or rhizosphere of different plant species. After 6 weeks, 5 g of fallow soil or roots with adhering soil were treated with 15 mL sterile tap water in a Stomacher blender (Seward, London, UK) at high speed for 60 s to release the microbes into the suspension. Fallow soil and rhizosphere suspensions were decanted from Stomacher bags. Suspensions were centrifuged for 5 min at 4000 g to obtain microbial pellets with remaining soil particles. The pellets were resuspended in sterile tap water and centrifuged for 5 min at 500 g to separate microbial suspensions from pelleted soil particles. The supernatant containing released soil microbes was sieved through a sterile 5‐μm sieve to remove native nematodes from the suspended microbiome. In a 15‐mL tube containing 5 mL microbial suspensions, 20,000 surface‐sterilised nematodes were incubated overnight at 22°C on a shaker at 150 rpm. Each treatment was replicated four times. As a control, nematodes were incubated in 5 mL sterile tap water. The nematodes were collected on 5‐μm sieves and washed with 10 mL sterile tap water to remove loosely adhering microbes from the surface of the nematodes. Washed nematodes were transferred from the sieve to bead‐beating tubes for DNA extraction to analyse the microbiomes on the cuticle of the nematodes.

#### 2.2.3 DNA Extraction and Next‐Generation Sequencing

The total community DNA of microbes associated with *P. penetrans* was extracted using the Fastprep FP120 bead beating system for 30 s at high speed and a FastDNA Spin Kit for Soil (MP Biomedicals, Heidelberg, Germany). To characterise the bacteria associated with the extracted nematodes, the V3–V4 regions of 16S rRNA genes were amplified using the primers 341F (5′‐CCTAYGGGRBGCASCAG‐3′) and 806R (5′‐GGACTACNNGGGTATCTAAT‐3′) (Caporaso et al. 2011; Sundberg et al. 2013) in a 25 μL reaction volume containing 2.5 μL of 10× reaction buffer (New England Biolabs, Frankfurt, Germany), 0.125 μL of 5 U/μL NEB HotStart Taq polymerase, 2.5 μL of 2 mM dNTP, 1 μL of 2.5 mM MgCl2, 2.5 μL of 2 mg/mL BSA, 1 μL of each primer (10 μM), and 1 μL of nematode DNA. The following temperature steps were applied: 2 min at 94°C, 30 cycles of 20 s at 94°C, 20 s at 56°C, 40 s at 72°C, followed by a final elongation for 5 min at 72°C. Amplicon sequencing of the 16S rRNA genes was done by 2 × 250 bp paired‐end high‐throughput sequencing on an Illumina HiSeq 2500 platform by Novogene (Cambridge, UK).

#### 2.2.4 Sequence Analysis

Processing of the bacteria sequence reads included paired‐end merging with an overlapping minimum read length of 10 base pairs and a minimum merge length of 400 bp using FLASH (Magoč and Salzberg 2011). Sequences with a low‐quality score were excluded (*Q* < 20; consecutive low‐quality bases of more than three or consecutive high‐quality base calls less than 75%). The remaining sequences were denoised, and chimeric sequences were removed using the Divisive Amplicon Denoising Algorithm (DADA2) (Callahan et al. 2017), as implemented in the Quantitative Insights Into Microbial Ecology 2 (QIIME 2) pipeline (Caporaso et al. 2010). The reads were annotated with classify‐sklearn (*p*‐confidence 0.7). Amplicon Sequence Variants (ASVs) were taxonomically assigned based on the SILVA database v. 138 for bacterial and archaeal 16S rRNA genes (Quast et al. 2013). Singletons, contaminants based on negative control, and non‐target reads (chloroplasts and mitochondria) were removed. ASVs with fewer than 10 reads across the entire data set were excluded from further analyses.

#### 2.2.5 Data Analysis

The counts of nematodes that invaded the plants were analysed using one‐way analysis of variance (ANOVA) to assess differences in nematode numbers between the treatment groups. Tukey's multiple comparisons test, with a single pooled variance, was performed to identify significant pairwise differences (GraphPad Prism version 10.0).

The multivariate analyses on the nematode‐associated bacterial ASV abundances were carried out with the R software version R3.6.3 (R Core Development Team) using the packages vegan (Oksanen et al. 2015), labDSV (Roberts 2016), and mvabund (Wang et al. 2012). All statistical analyses were done on non‐rarefied ASV data, but normalisation of sequencing depth was based on relative abundances (i.e., sequence counts in each column were scaled by the column's sum). Diagnostic plots of residuals versus fitted values revealed the lack of significant heterogeneity of variance, and Q–Q plots showed that assumptions of normality were justified. To test the effect of the treatment, suppressiveness, and the compartment on the changes in the nematode‐associated bacterial communities, a permutational multivariate analysis of variance (PERMANOVA) was used (Anderson 2001). The PERMANOVA analysis was based on Bray–Curtis dissimilarity matrices using 10,000 permutations calculated from logarithmically transformed data. Differences between community compositions were visualised using Principal Coordinate Analysis (PCoA). The method is based on Bray–Curtis dissimilarities and was performed with logarithmically transformed relative abundance data. The squared multiple correlation coefficient *R* ^2^, as an effect measure, can be interpreted as the proportion of variability in the observed similarity measures explained by the factor tested. Random Forest regression was used to identify and predict differentially abundant ASVs that best explained differences in suppressiveness (Breiman 2001). Liaw and Wiener (2002) modelled the relationships between microbial ASVs and plant‐nematode traits (nematode count, number of nematodes invading the root, and root weight). Feature matrices were constructed by combining selected ASVs with relevant metadata. Models were trained on 70% of the data and tested on the remaining 30%, with performance assessed using root mean square error (RMSE) and coefficient of determination (*R* ^2^). Feature importance was evaluated based on the increase in mean squared error (Breiman 2001; Liaw and Wiener 2002). The top predictive features for nematode count were further analysed using Spearman's rank correlation, and results were visualised as correlation matrices. ASV identifiers were annotated to the genus level to enhance biological interpretation.

## 3 Results

### 3.1 Plant Species Influence *P. penetrans* Microbiome Association and Invasion Into Barley

**FIGURE 2:**
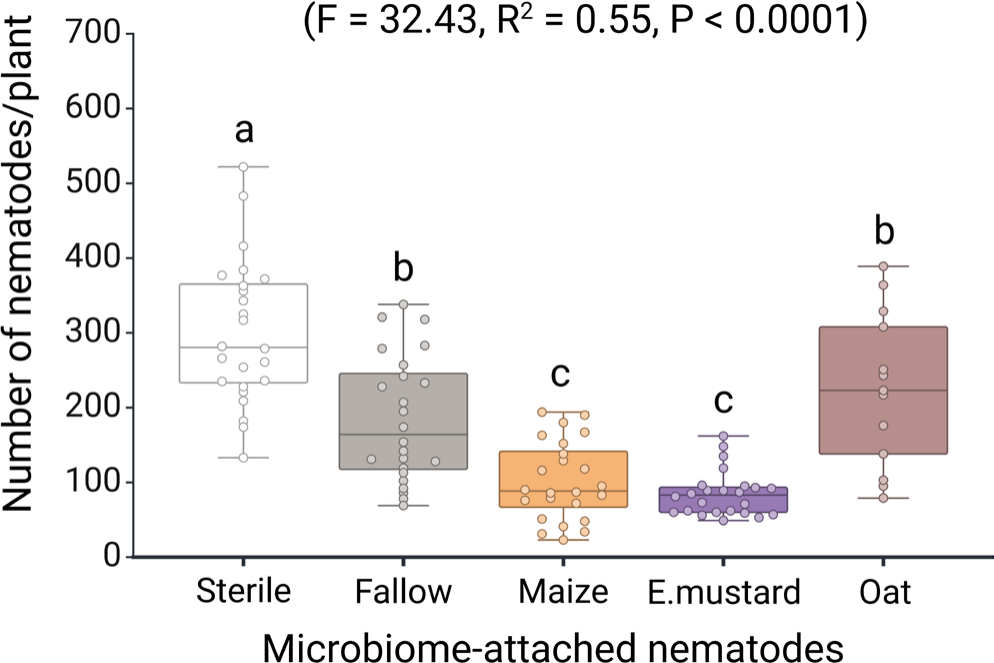
Effect of *P. penetrans* attached microbiomes originating from the rhizospheres of different plant species on their invasion into barley roots. *P. penetrans* were baited in microbiomes from fallow soil, maize, Ethiopian mustard, or oat rhizosphere soil; control: Surface‐sterilised nematodes (*n* = 24). Boxplots show the median (horizontal line), interquartile range (box), and the minimum and maximum values within 1.5× the interquartile range (whiskers); individual points represent biological replicates. Statistical differences between treatments were assessed using Tukey's HSD post hoc test; groups labelled with different letters differ significantly (*p* < 0.05).

We exposed surface‐sterilised *Pratylenchus penetrans* to suspensions from various rhizospheres to test whether the microbiome source alters the nematode‐associated community and, in turn, their ability to invade barley roots. The type of microbiome significantly affected the number of nematodes that invaded the roots. The nematodes baited in microbial suspensions derived from fallow soil or soils conditioned by Ethiopian mustard, maize, or oat exhibited notable variations in their invasion into barley roots (*F* = 32.43, *R* ^2^ = 0.55, *p‐*value < 0.0001) (Figure 2). The invasion rates of *P. penetrans* baited in fallow soil or rhizosphere microbiomes were significantly lower compared to *P. penetrans* without attached microbiome. Baiting in the oat rhizosphere microbiome and fallow soil microbiome did not significantly differ in effect on root invasion of *P. penetrans* (*p*‐value = 0.54). The most potent inhibition of *P. penetrans* invasion was observed through their association with the microbiomes of maize and Ethiopian mustard (Table S2). In contrast, the nematode's association with the oat microbiome failed to reduce the invasion of *P. penetrans* into barley roots. Our findings suggest that, beyond genetic resistance to nematodes, rhizosphere microbes can strongly influence the core microbiome attached to the cuticle, thereby impacting nematode invasion into host plant roots.

### 3.2 Plant‐Driven Shifts in Cuticle‐Associated Microbiomes of *P. penetrans*

We explored the extent to which the observed effects on *P. penetrans* invasion are linked to the shift in the composition of the cuticle‐attached bacteria between the soil microbiomes that different plant species have conditioned. Overall, the effect of plant species that condition the soil microbiome on the suppression of *P. penetrans* is reflected by the bacterial community composition on the cuticle. The composition of the bacterial community associated with the nematode's cuticle is notably influenced by compartment (soil vs. rhizosphere) and treatment (plant species). The comparison between rhizosphere microbiomes and the fallow soil microbiome accounted for the largest share of variance in bacterial communities attached to the cuticle of *P. penetrans* (*R* ^2^ = 0.258, *p* < 0.001) (Table 1). Similarly, the plant species that shaped the microbiome had a significant influence on shaping the bacterial community associated with the nematode cuticle, accounting for a similar portion of the variance (*R* ^2^ = 0.237, *p* < 0.001). Meanwhile, the composition of this bacterial community significantly contributed to *P. penetrans* suppression but explained less variation compared to the compartment and plant species factors (*R* ^2^ = 0.18, *p* < 0.001). These results suggest that a subset of bacteria filtered by the nematode cuticle from the plant's rhizosphere plays an important ecological role.

**TABLE 1:** Permutational multivariate analysis of variance (PERMANOVA) of bacterial community composition on the surface of root‐lesion nematodes in soil type B, testing the effects of compartment (fallow soil vs. rhizosphere soil), suppressiveness (Ethiopian mustard, maize vs. oat, fallow), and treatment (baiting in fallow‐soil, maize, Ethiopian mustard, or oat rhizosphere microbiomes).

Fixed effect	*F*	*R* ^2^	*p*
Compartment	9.67	0.258	0.001
Suppressiveness	6.86	0.18	0.001
Treatment	8.86	0.237	0.001

**FIGURE 3:**
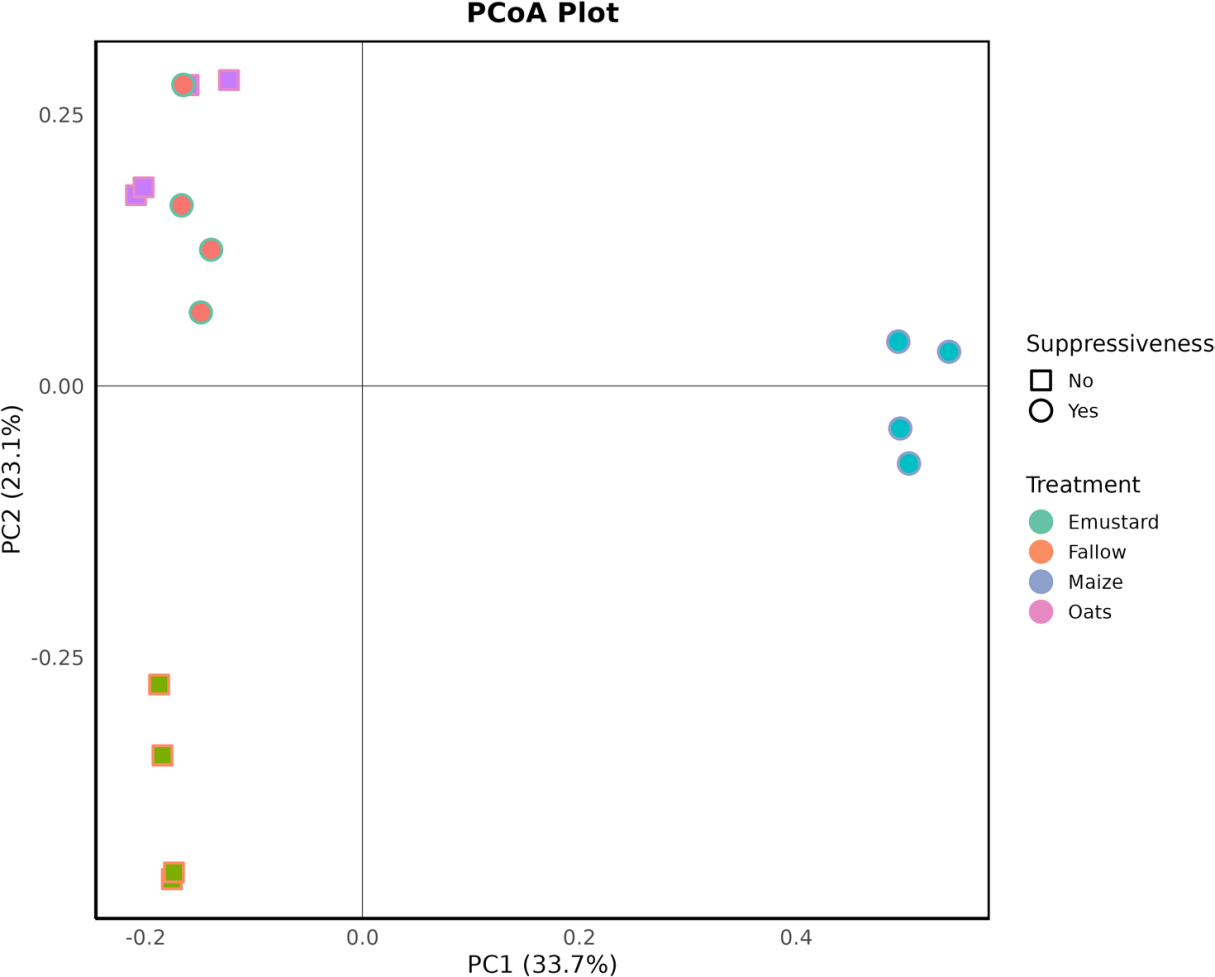
Principal Coordinates Analysis (PCoA) of bacterial communities associated with nematode cuticles across different rhizosphere and fallow soils. The plot depicts variations in bacterial community composition based on treatment (Ethiopian mustard, fallow, maize, and oats) and their associated suppressiveness toward root‐lesion nematodes. The percentage variance explained by each principal coordinate axis is shown in parentheses.

The principal coordinates analysis showed that the bacterial community associated with the cuticle of *P. penetrans* after baiting in the maize rhizosphere microbiome is highly different from that after baiting in rhizosphere microbiomes from the other plant species (Figure 3). The difference is well represented by PC1, which explained 33.7% of the total variance. Despite a similar suppressive effect of cuticle‐associated bacteria conditioned by maize and Ethiopian mustard, the composition of the bacterial community largely differed. Instead, the bacterial community on the nematodes after baiting in the Ethiopian mustard rhizosphere microbiome was much more similar to that after baiting in the non‐suppressive oat rhizosphere microbiome (Figure 3). The structure of bacterial communities on the cuticle after baiting in fallow soil microbiome differed from that after baiting in rhizosphere microbiomes in both PC1 (maize) and PC2 (Table 2).

**TABLE 2:** Bacterial ASVs on the cuticle of *P. penetrans* that were significantly associated in their relative abundance with reduced nematode counts in the roots of barley, and their taxonomic affiliation.

ASV	Bacterial family	Genus	*R* ^2^	*p*
ASV_75	Weeksellaceae	*Chryseobacterium*	0.371	0.028
ASV_312	Streptomycetaceae	*Streptomyces*	0.4164	0.014
ASV_120	Burkholderiaceae	*Duganella*	0.4072	0.02
ASV_171	Burkholderiaceae	*Duganella*	0.434	0.014
ASV_79	Enterobacteriaceae	*Enterobacteriaceae*	0.3724	0.039
ASV_323	Caulobacteraceae	*Asticcacaulis*	0.349	0.05
ASV_94	Burkholderiaceae	*Acidovorax*	0.3475	0.045
ASV_113	Bacillaceae	*Bacillus*	0.3619	0.05
ASV_241	Soil bacterium WF55	Soil bacterium WF55	0.3375	0.05
ASV_151	Rhodanobacteraceae	*Luteibacter*	0.4065	0.027
ASV_215	Rhodanobacteraceae	*Rhodanobacter*	0.4507	0.009
ASV_125	Rhodanobacteraceae	*Dyella*	0.5697	0.003
ASV_246	Pseudomonadaceae	*Pseudomonas*	0.4444	0.008
ASV_82	Burkholderiaceae	*Burkholderia‐Caballeronia‐Paraburkholderia*	0.6071	0.002
ASV_110	Sphingobacteriaceae	*Mucilaginibacter*	0.5573	0.005
ASV_213	Burkholderiaceae	*Burkholderia‐Caballeronia‐Paraburkholderia*	0.7456	0.001
ASV_108	Pseudomonadaceae	*Pseudomonas*	0.7915	0.001

*Note:* *R* ^2^ indicates how much of the variation in nematode counts is explained by the abundance of each ASV, while the *p*‐value shows the statistical significance of this association (with values < 0.05 considered significant).

**FIGURE 4:**
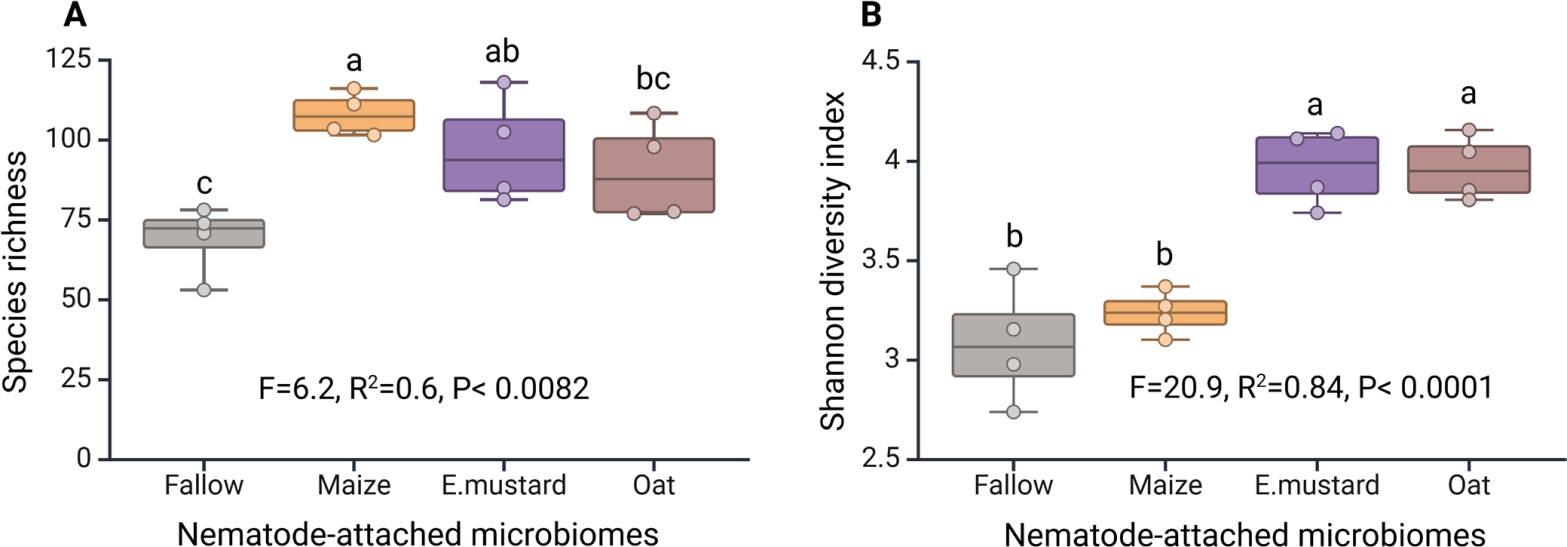
Diversity of bacterial communities on the surface of root‐lesion nematodes after baiting in microbial suspensions from fallow soil or the rhizospheres of maize, Ethiopian mustard, or oat. Different letters indicate significant differences in a Tukey test (*n* = 4).

The bacterial species richness associated with the nematode cuticle differed significantly between suspensions derived from the various rhizosphere and fallow microbiomes (ANOVA, *F* = 6.2, *R* ^2^ = 0.6, *p* = 0.009) (Figure 4). Specifically, the nematode exposed to the maize microbiome had higher bacterial species richness on the cuticle compared to both fallow and oat microbiomes. Conversely, the treatment by the Ethiopian mustard microbiome demonstrated a comparable effect to the oat microbiome but exhibited significantly higher species richness compared to the treatment by the fallow soil microbiome. The bacterial diversity, as indicated by the Shannon index, was significantly higher when *P. penetrans* was exposed to Ethiopian mustard and oat microbiomes compared to maize and fallow soil microbiomes. The phylum Proteobacteria was the most abundant on the *P. penetrans* cuticle. However, on the cuticle of *P. penetrans* incubated in the maize microbiome, Proteobacteria were relatively abundant in combination with Firmicutes (Figure S1).

### 3.3 Suppressive Species Demonstrated Host‐Specificity and Shared Presence Across Different Plant Species

**FIGURE 5:**
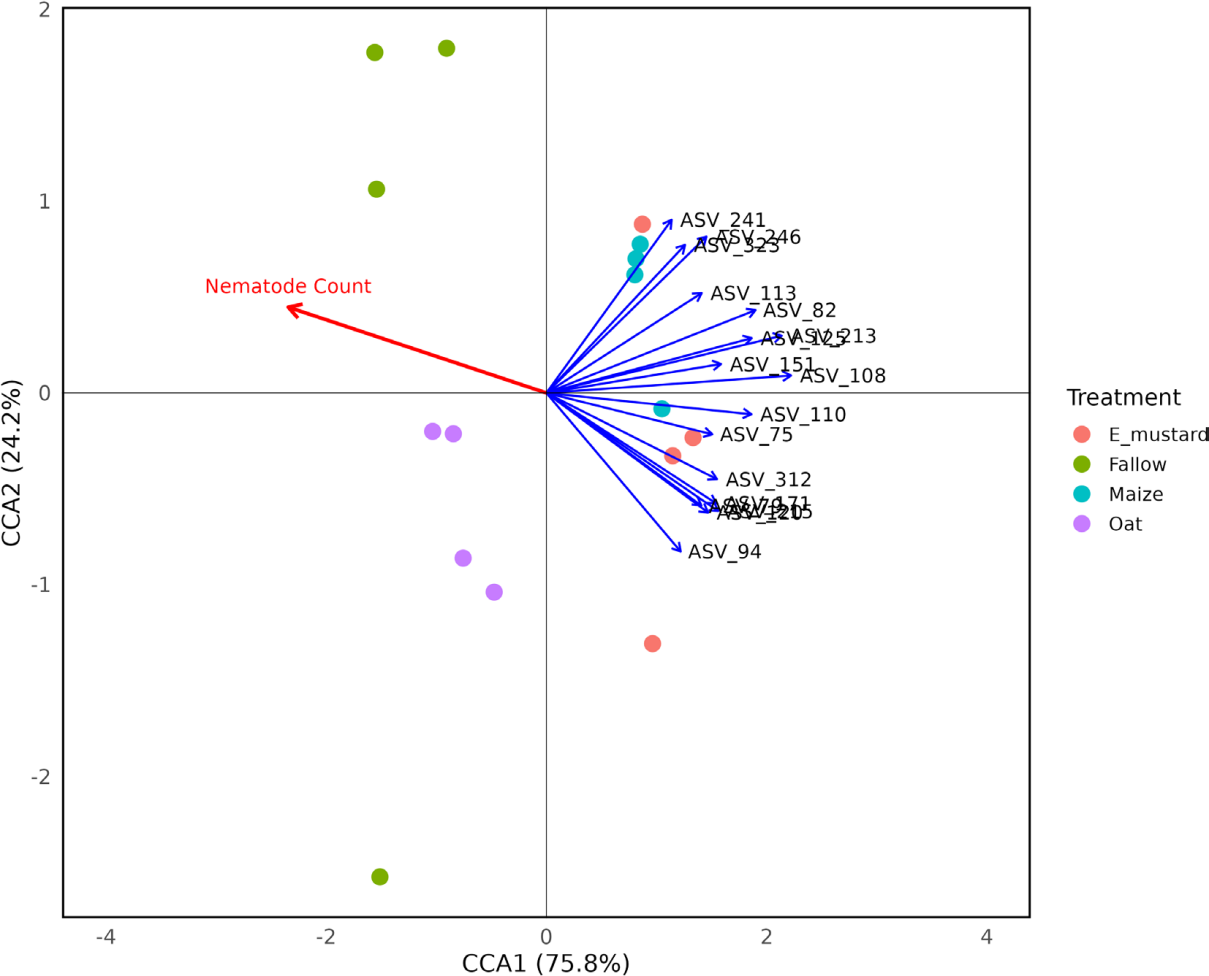
Canonical correspondence analysis (CCA) illustrates the relationship between bacterial species associated with the cuticle of *P. penetrans* and nematode counts in the roots of barley plants. The indicated amplicon sequence variants (ASVs) showed a negative correlation with nematode counts in the barley root system.

**FIGURE 6:**
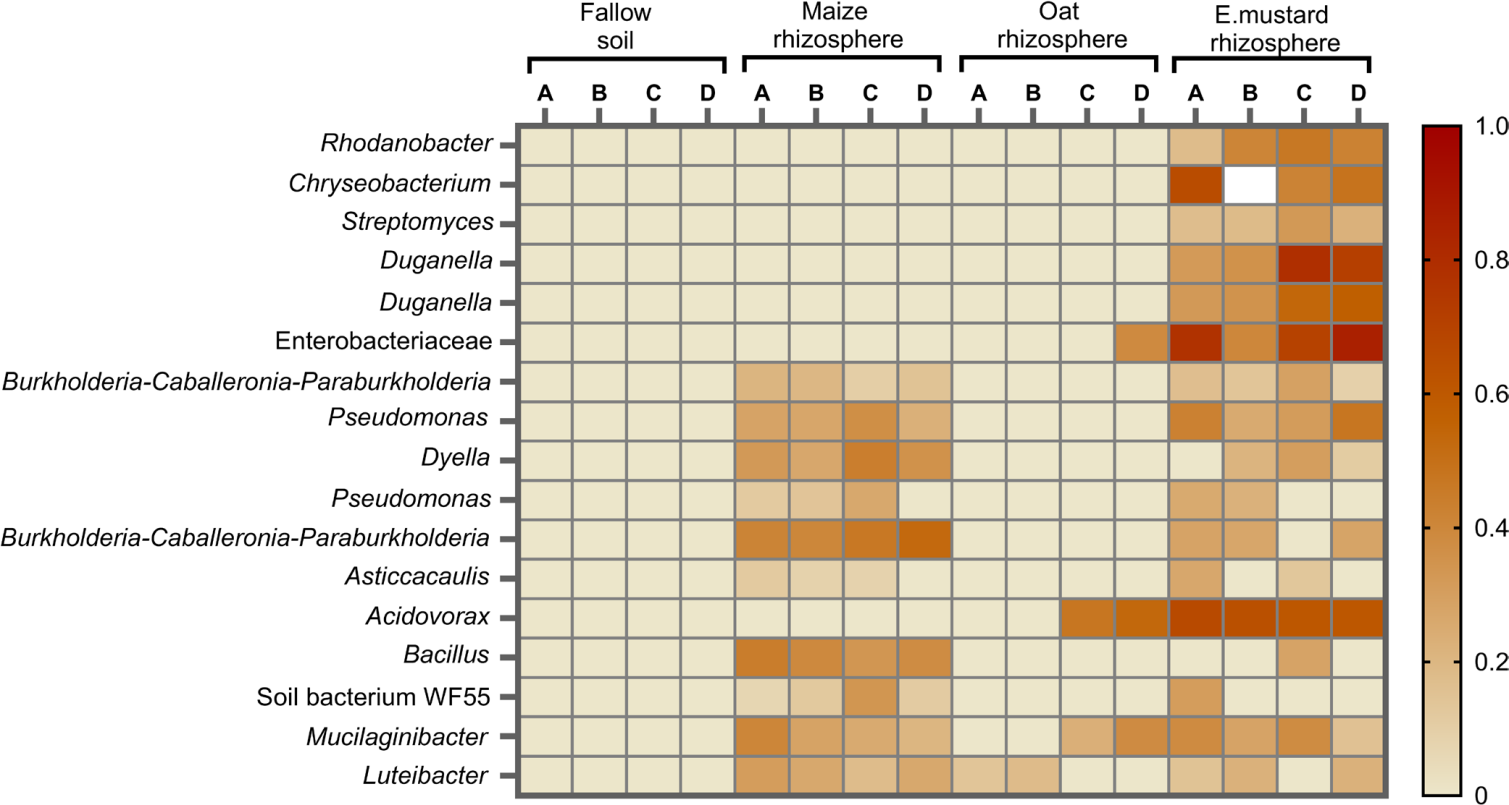
Heatmap of the relative abundances of bacterial ASVs associated with the *P. penetrans* cuticle, which were significantly associated with nematode counts in the roots of barley, across different soil and rhizosphere microbiomes in which the nematodes were baited. Each column (A, B, C, D) represents biological replicates for each treatment. Bacterial species abundance scores are depicted using a gradient colour scale, where beige indicates no abundance and dark red indicates the saturation threshold.

**FIGURE 7:**
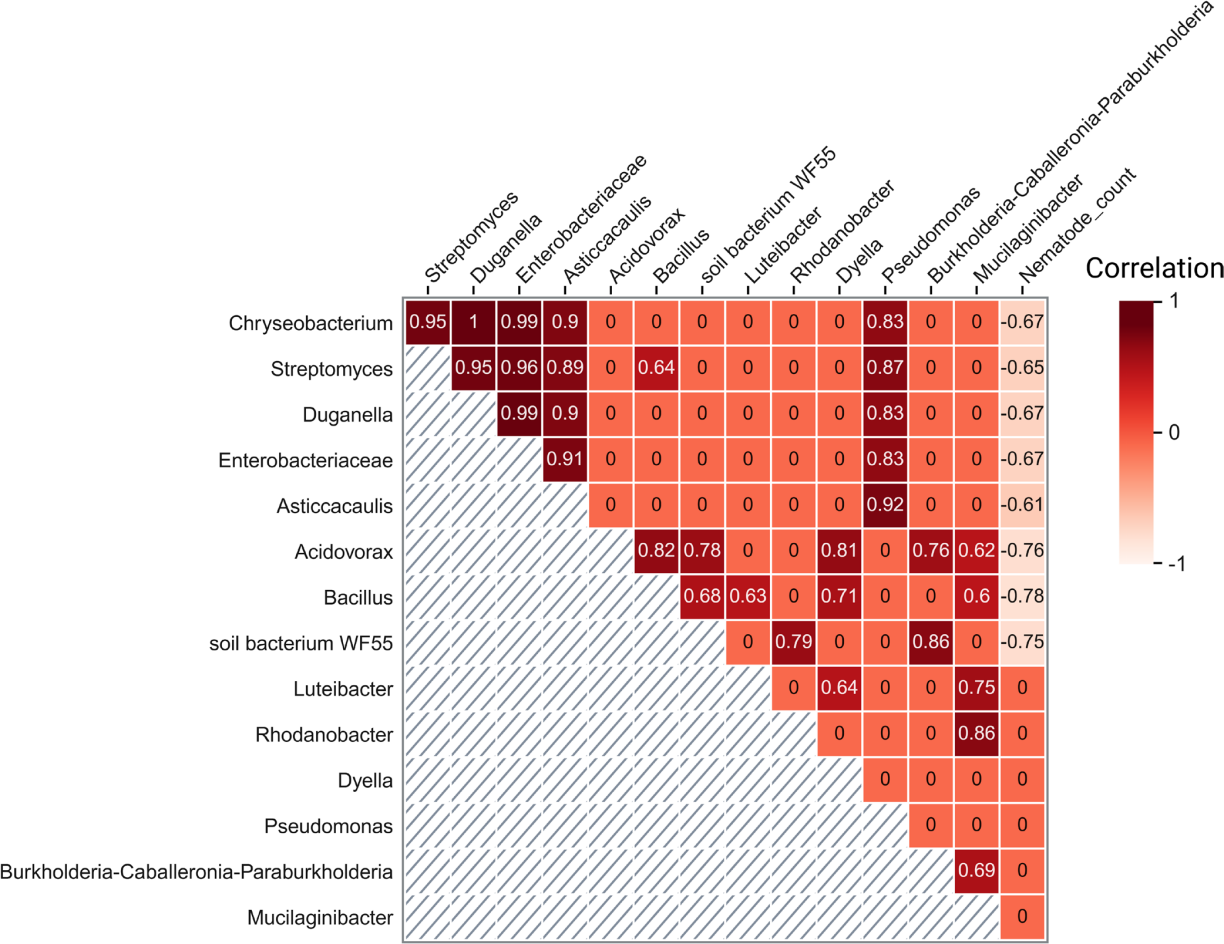
Correlation analysis of bacterial ASVs and nematode count in the barley roots. The heatmap shows strong positive correlations between bacterial ASV, which indicates bacterial coexistence on the nematode cuticle (squares with red colour), and is negatively correlated with nematode invasion into plant roots (squares with blue colour).

Certain suppressive bacterial species exhibited a remarkable degree of host specificity; however, other species shared presence across nematode cuticle exposed to the different plant species microbiomes (Figures 5 and 6). Multivariate analyses, including CCA and permutational tests, were employed to explore the relationship between ASVs and the number of nematodes in the barley roots. The first axis of the CCA (CCA1), explaining 76% of the variance, clearly distinguished bacterial communities associated with the nematode cuticle by their ability to suppress nematodes. This separation highlighted the prevalence of suppressive bacterial ASVs in the rhizospheres of maize and Ethiopian mustard. The second axis (CCA2) explained an additional 24% of the variance, which revealed that specific nematode‐suppressive ASVs were uniquely associated with individual host plants, either maize or Ethiopian mustard. These findings showed host‐specific interactions between suppressive bacterial communities and nematodes, potentially influenced by the distinct conditioning of soil by each plant within its rhizosphere. These suppressive bacterial ASVs predominantly belonged to Alphaproteobacteria, Betaproteobacteria, and Gammaproteobacteria, with additional representatives from Actinomycetia, Bacilli, Flavobacteria, and Sphingobacteria. Burkholderiaceae and Rhodanobacteraceae emerged as the most common bacterial families that comprise suppressive ASVs closely associated with nematodes in maize and Ethiopian mustard rhizospheres. Of particular interest were the highly significant suppressive species abundant on nematode cuticles, including ASVs of Burkholderia‐Caballeronia‐Paraburkholderia (ASV82, *R* ^2^ = 0.6, *p* < 0.002; ASV213, *R* ^2^ = 0.74, *p* < 0.001) and *Pseudomonas* (ASV108, *R* ^2^ = 0.79, *p* < 0.001; ASV246, *R* ^2^ = 0.44, *p* < 0.002). *Mucilaginibacter*, *Rhodanobacter*, and *Dyella* also demonstrated significant associations with *P. penetrans* suppression. Analysis of suppressive ASVs revealed that all species found in the maize rhizosphere were also present in the rhizosphere of Ethiopian mustard. However, certain ASVs exhibiting significant suppressive effects on nematodes were unique to the Ethiopian mustard rhizosphere. Notable taxa on nematode baited in the Ethiopian mustard rhizosphere included *Chryseobacterium* (ASV75), *Streptomyces* (ASV312), *Duganella* (ASV120, ASV171), Enterobacteriaceae (ASV79), *Acidovorax* (ASV94), and Rhodanobacter (ASV215). In contrast, soil bacterium WF55 (ASV241) and *Bacillus* (ASV113) occurred exclusively on nematode baited in the maize rhizosphere and correlated with nematode suppression. On the other hand, correlation analysis revealed strong positive associations among specific bacterial ASVs, which indicate bacterial co‐existence within the core microbiome on the nematode cuticle or reflect shared ecological niches. Meanwhile, this coexistence demonstrated a negative relationship with the number of nematodes invading barley roots. For instance, *Chryseobacterium*, *Duganella*, *Streptomyces*, *Asticcacaulis*, *Pseudomonas*, and Enterobacteriaceae exhibited high positive correlations in their coexistence (> 0.83) and were associated with reduced nematode root invasion (Figure 7). Similarly, *Bacillus* and *Acidovorax* exhibited a positive correlation with *Mucilaginibacter*, *Burkholderia‐Caballeronia‐Paraburkholderia*, the soil bacterium WF55, and *Dyella* (> 0.60) while negatively correlating with nematode numbers in the roots.

## 4 Discussion

The association between plants and their microbiomes has emerged as a valuable tool for supporting plant functional traits, either through transplanting or enriching processes that lead to diverse or core‐filtering microbial communities (Mukherjee et al. 2012; van der Heijden and Schlaeppi 2015; Wagg et al. 2019; Suman et al. 2022). In our study, the core microbiome filtered by *P. penetrans* determined its invasion into the root system of barley plants. The rhizosphere microbiome, enriched with plant‐specific taxa and contacted by infective stages before invasion, determines the number of nematodes that enter roots. This suggests that, beyond plant genetic resistance to nematodes, enriched rhizosphere microbes play a crucial role in influencing the core microbiome attached to the nematode cuticle, impacting the nematode's ability to invade host plant roots. The differential capacity of soil microbiomes between soil types in suppressing plant–parasitic nematodes was previously shown (Adam et al. 2014; Topalović et al. 2020). However, the impact of the rhizosphere soil microbiome from different plant types or species on nematode suppression has been rarely investigated. Compared with surface‐sterilised nematodes, microbiomes from both fallow and rhizosphere soils significantly reduced nematode invasion into barley roots. Maize and Ethiopian mustard microbiomes were the most effective in inhibiting *P. penetrans* invasion, whereas the oat microbiome did not reduce nematode invasion into barley roots. This suggests that both the soil microbiome and the specific plant species interact to influence nematode invasion into barley roots. This could be attributed to the fact that the root‐associated microbiome is tightly connected to plant genetic factors, forming a “holobiont” that contributes to nematode suppression and probably provides other functions for the host plant (Hacquard 2016; Hacquard et al. 2017; Sánchez‐Cañizares et al. 2017; Hassani et al. 2018).

The suppressive effect of the bacterial community associated with nematode cuticles was significantly correlated with the composition of these communities. Interestingly, the relationship between bacterial diversity, species richness, and nematode suppression varied depending on the plant species. Specifically, the suppressive maize microbiome exhibited the highest bacterial species richness on the nematode cuticle but not in the rhizosphere soil. This suggests that certain bacterial species may play a key role in nematode suppression due to their abundance. Conversely, Ethiopian mustard had both higher species richness and diversity and concomitantly was effective in suppressing *P. penetrans* . Furthermore, our findings showed that the suppression of *P. penetrans* was related not only to bacterial species conserved across different plant species, such as maize and Ethiopian mustard, but also to specific bacterial species associated with each plant species. Notably, species of *Rahnella*, *Burkholderia‐Caballeronia‐Paraburkholderia*, *Pseudomonas*, *Mucilaginibacter*, and *Luteibacter* were enriched on *P. penetrans* recovered from the rhizosphere of both maize and Ethiopian mustard. Meanwhile, ASV_113, related to *Bacillus*, and ASV_241, related to a soil bacterium WE55, exhibited specificity to maize. In contrast, *Chryseobacterium*, *Streptomyces*, *Duganella*, Enterobacteriaceae, *Rhodanobacter*, and *Acidovorax* exhibited specificity to Ethiopian mustard.

Previously, a consortium of *Bacillus licheniformis* and *Pseudomonas fluorescens* isolated from the egg masses and second‐stage juveniles of root‐knot nematodes showed great potential in reducing the gall formation on tomato roots (Colagiero et al. 2018). Some other species represent different mechanisms to suppress nematodes; for example, *Burkholderia* sp. can restrain the nematodes outside the root system of the host plant (Liu et al. 2022), secrete lipases to control nematodes like *Bursaphelenchus xylophilus*, release HCN gaseous molecules that inhibit the mitochondrial apparatus (Gallagher and Manoil 2001), or directly antagonise or induce systemic resistance toward nematode infection. Our findings suggest that the soil microbiome conditioned by different pre‐crops can have a significant impact on the suppression of *P. penetrans* invasion. Some plant species and genotypes have a positive legacy impact on nematode suppression, while others are more susceptible to *P. penetrans* invasion and do not develop a filtered core of microbiota. For instance, microbiomes conditioned by Ethiopian mustard and maize showed significant suppression compared to the unconditioned microbiome of fallow soil. These results highlight the importance of considering plant species in the selection of crops for nematode suppression in agricultural practices, not only because of their resistance but also their ability to harness key suppressive strains in the rhizosphere. Identifying the dominant nematode species and integrating the proper crop in the crop rotation was also evident in our findings, as the invasion of nematodes was significantly reduced with maize and Ethiopian mustard microbiomes. Both plants and associated microbes in the soil play a significant role in nematode suppression through different mechanisms. Root exudates, regardless of their direct effect on nematodes, the secreted exudates from the mycorrhizal colonised plants attract other beneficial microbial species like *Pseudomonas fluorescens* and *Trichoderma* spp., which exhibit nematicidal properties for biological control of nematodes (Gupta Sood 2003; Druzhinina et al. 2011). Beneficial microbes that attach to the nematode's surface coat, like fungi and bacteria, can be studied in greater depth by identifying the genes regulated in the nematode's presence to understand the mechanisms underlying the suppression. Understanding the exchanged signals among nematodes, plants, and microbes might direct the efforts much better to find a specific solution or new nematicidal effects.

## 5 Conclusion

Our study highlights the significant role of plant–soil microbiome interactions in suppressing the invasion of *P. penetrans* into barley roots. The composition and diversity of microbial communities, influenced by specific plant species, are crucial in determining the nematode suppression capacity of rhizospheres. Microbiomes conditioned by maize and Ethiopian mustard were particularly effective in inhibiting nematode invasion, with plant‐specific bacterial species playing a key role in this suppression. These findings show the importance of integrating plant species selection and microbial community management in agricultural practices to enhance nematode suppression. In addition to assembling microbial communities, optimising crop rotation and pre‐cropping strategies can steer plant‐associated microbiomes to reduce nematode damage. Future research should focus on exploring the molecular mechanisms underlying microbial coexistence and interactions to develop more targeted, sustainable approaches for nematode management in agriculture.

## Author Contributions

A.E., H.H. and X.K. conceptualised and outlined the structure of the study. A.E. and S.A. conducted experiments. A.E. wrote the first draft of the manuscript, and A.E. and X.K. designed the figures. X.K. analysed the microbiome attached to the nematode cuticle. A.E., X.K., S.A., and H.H. revised the manuscript and approved the final version for submission.

## Conflicts of Interest

The authors declare no conflicts of interest.

## Supporting information


**Figure S1:** Taxonomic profiles of bacterial communities on the surface of *P. penetrans* after baiting in microbial suspensions from fallow soil or the rhizospheres of maize, Ethiopian mustard, or oat. The relative abundances are shown on the phyla level (*n* = 4).
**Table S1:** Physical and chemical properties of the different soil types used to produce the tested microbiomes from the different plant species
**Table S2:** Pairwise comparisons showing the effects of soil type and plant species on *P. penetrans* microbiome association and invasion into barley roots. Statistical significance was assessed by one‐way ANOVA followed by multiple comparison tests. Significance indicated by asterisks (****p < 0.0001, ns = not significant).

## Data Availability

The data that supports the findings of this study are available in the SI of this article.
