# Supplementary material for: Pre‐Crop Choice Shapes Nematode‐Attached Bacterial Communities Associated With Reduced Pratylenchus penetrans Invasion of Barley Roots
Source: Environ Microbiol. 2025 Sep 18;27(9):e70179. doi: 10.1111/1462-2920.70179 (PMC12445950; doi:10.1111/1462-2920.70179)
Supplement: Supplementary file 1 — Figure S1: Taxonomic profiles of bacterial communities on the surface of P. penetrans after baiting in microbial suspensions from fallow soil or the rhizospheres of maize, Ethiopian mustard, or oat. The relative abundances are shown on the phyla level (n = 4). Table S1: Physical and chemical properties of the different soil types used to produce the tested microbiomes from the different plant species Table S2: Pairwise comparisons showing the effects of soil type and plant species on P. penetrans microbiome association and invasion into barley roots. Statistical significance was assessed by one‐way ANOVA followed by multiple comparison tests. Significance indicated by asterisks (****p < 0.0001, ns = not significant). [file EMI-27-e70179-s001.docx]

**Pre-crop choice shapes nematode-attached bacterial communities associated with reduced *Pratylenchus penetrans* invasion of barley roots**

Ahmed Elhady^1,2,3*^, Xorla Kanfra^1^, Shimaa Adss^1^ , Holger Heuer^1*^

^1^Institute for Epidemiology and Pathogen Diagnostics, Julius Kühn-Institute (JKI) - Federal Research Centre for Cultivated Plants, Braunschweig, Germany

^2^Darwin21 Desert Research Initiative, Biological and Environmental Science and Engineering Department, King Abdullah University of Science and Technology, Thuwal, Saudi Arabia

^3^Department of Plant Protection, Faculty of Agriculture, Benha University, Benha, Egypt

*Correspondence:

[ahmed.elhady@kaust.edu.sa](mailto:ahmed.elhady@kaust.edu.sa); [Holger.Heuer@julius-kuehn.de](mailto:Holger.Heuer@julius-kuehn.de)

**Supplementary**

**Figure S1**


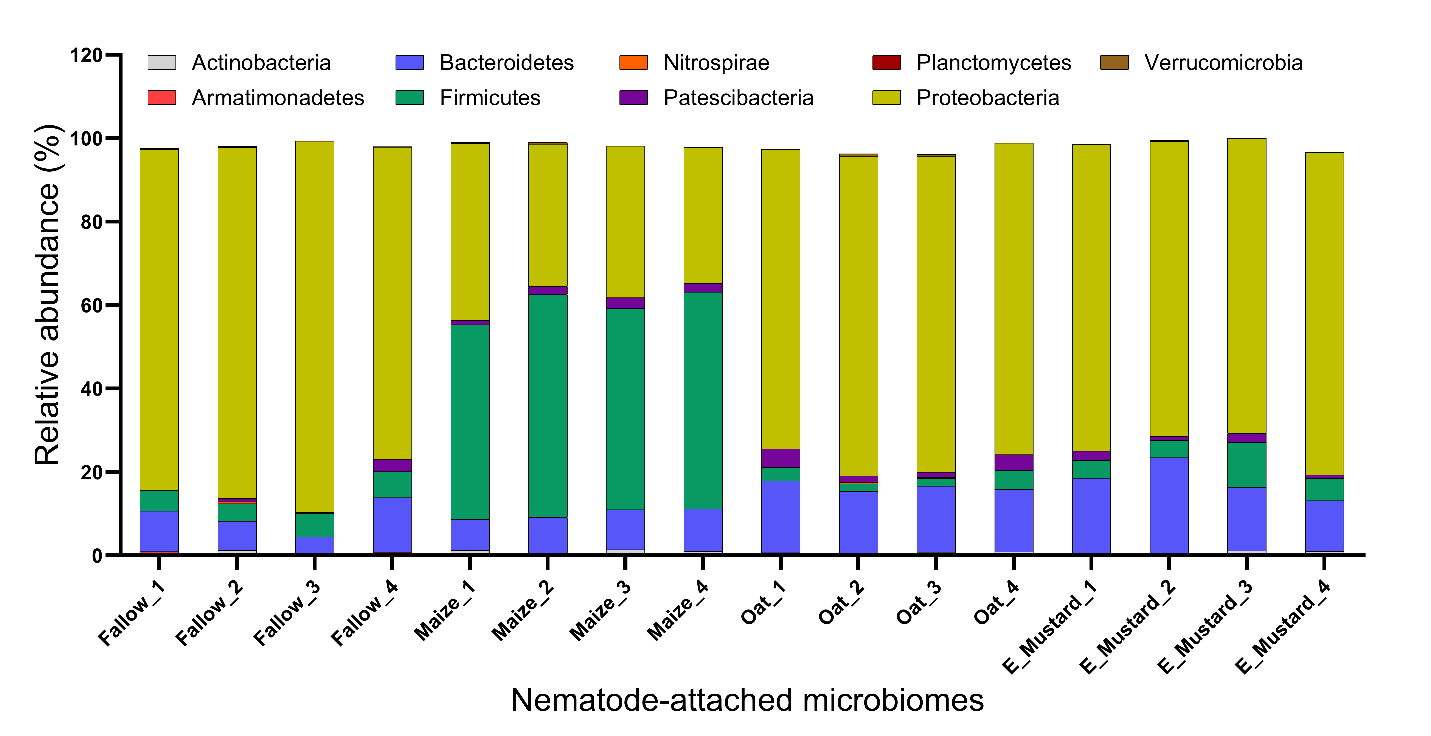


Figure S1. Taxonomic profiles of bacterial communities on the surface of *P. penetrans* after baiting in microbial suspensions from fallow soil or the rhizospheres of maize, Ethiopian mustard, or oat. The relative abundances are shown on the phyla level (n=4).

**Table S1**: Physical and chemical properties of the different soil types used to produce the tested microbiomes from the different plant species

| Soil sample | | Braunschweig JKI |
| --- | --- | --- |
| Soil usage | | Acker |
| Soil type | | Sandy silt |
| Soil classification | | (h)S |
| Lime pH | CaCl₂ | 6.3 E |
| Phosphorus (P) mg in | 100g of soil | 7.2 C |
| Potassium (K)mg in | 100g of soil | 28.6 E |
| Magnesium (mg) mg in | 100g of soil | 3.8 B |
| Clay % | | 4.70% |
| Silt % | | 25.30% |
| Sand % | | 70.00% |
| Soil type Abbr. (KA 5) | | Su3 |
| Carbon (Corg.) | | 0.58% |
| Total Nitrogen (N) | | 0.05% |
| C/N ratio | | 12 |
| Humus content | | 1.00% |

**Table S2.** Pairwise comparisons showing the effects of soil type and plant species on P. penetrans microbiome association and invasion into barley roots. Statistical significance was assessed by one-way ANOVA followed by multiple comparison tests. Significance indicated by asterisks (****P < 0.0001, ns = not significant).

| **Comparison** | **P-value** | **Asterisk** | **Difference between means** |
| --- | --- | --- | --- |
| Sterile vs Fallow | 0.03498 | * | 26.6458 |
| Sterile vs Maize | <0.0001 | **** | 54.6875 |
| Sterile vs E. mustard | <0.0001 | **** | 61.7917 |
| Sterile vs Oat | 1 | ns | 16.8285 |
| Fallow vs Maize | 0.02118 | * | 28.0417 |
| Fallow vs E. mustard | 0.001173 | ** | 35.1458 |
| Fallow vs Oat | 1 | ns | -9.8173 |
| Maize vs E. mustard | 1 | ns | 7.1042 |
| Maize vs Oat | 0.005041 | ** | -37.859 |
| E. mustard vs Oat | 0.0003616 | *** | -44.9631 |
